# Uncommon Presentation of a Benign Brenner Tumor Mimicking Malignancy: A Case Report

**DOI:** 10.1155/crog/8794499

**Published:** 2026-09-15

**Authors:** Sulaeman Andrianto Susilo, Ali Budi Harsono, Hermin Aminah Usman

**Affiliations:** ^1^ Department of Obstetrics and Gynecology, Hasan Sadikin Central General Hospital, Universitas Padjadjaran, Bandung, Indonesia, unpad.ac.id; ^2^ Department of Pathology Anatomy, Hasan Sadikin Central General Hospital, Universitas Padjadjaran, Bandung, Indonesia, unpad.ac.id

**Keywords:** Brenner tumor, histopathology, immunohistochemistry, ovarian malignancy, ovarian tumor

## Abstract

**Background and Aims:**

Brenner tumor (BT) is a rare ovarian epithelial tumor, with approximately 95% of cases being benign. Because of its rarity and nonspecific clinical radiological features, benign BT may mimic ovarian malignancy. We present a case of a benign BT with clinical, radiological, and intraoperative findings suggestive of malignancy.

**Method:**

A 69‐year‐old postmenopausal woman presented with progressive abdominal enlargement, abdominal pain, and a 10 kg weight loss over 2 months. Ultrasonography showed an ovarian mass with both benign and malignant IOTA features, whereas risk of malignancy index‐2 (RMI‐2) was 1350. Abdominal laparotomy with total hysterectomy, bilateral salpingo‐oophorectomy, and omentectomy was performed. Histopathological examination of the ovarian mass demonstrated features suspicious for MBT, whereas epithelial cell clusters identified in the omental specimen raised concern for metastatic involvement. Immunohistochemical examination showed CK7 and p63 positivity in ovarian tumor but negative expression in omental specimen, excluding metastatic BT.

**Result:**

Despite the patient′s postmenopausal status, large tumor size, elevated CA‐125 level, high RMI‐2 score, and intraoperative omental adhesion, the final diagnosis was benign BT. The case demonstrates the potential for benign BTs to mimic advanced ovarian malignancy clinically and radiologically. Immunohistochemistry was particularly useful in resolving the diagnostic uncertainty regarding the suspected omental metastasis.

**Conclusion:**

Benign BTs may present with features strongly suggestive of malignancy. Careful histopathological evaluation is essential, and immunohistochemistry can provide valuable diagnostic clarification when metastatic involvement or malignant transformation is suspected.

## 1. Introduction

Brenner tumor (BT) is one of the rare histologic types of epithelial ovarian tumors that account for less than 3% of all ovarian neoplasms. It was first described by Dr. Fritz Brenner who had found 3 cases in 1907 and named them “oophoroma folliculare” with abnormally developed ovarian follicles that look like a variant of granulosa cell tumor [1]. There are no specific clinical or radiological characteristics of this condition but it usually unilateral and occurs in postmenopausal women [2]. Based on histological data, BT can be further classified as benign, borderline (proliferative), and malignant [3]. The most common BT type was benign, approximately 95% of cases, whereas borderline and malignant tumor account for < 5% and < 1%, respectively. BT was composed of transition like epithelial cell nests surrounded dense fibrous stroma, related to the normal transitional cell of the urinary tract.

It can be difficult to make the right pathology diagnosis of a BT. There may be overlap and subjectivity in the diagnostic criteria and differential diagnosis between the three categories (benign, borderline, and malignant). Important evidence for the diagnosis of a benign BT is provided by the presence of distinctive epithelial cell nests, dense fibromatous stroma, and significant cytological metaplasia without atypia. BTs that lack stromal invasion and have epithelial proliferation that surpasses that of benign BTs are classified as borderline BTs. Malignant BT (MBT) arises from benign and borderline tumors that exhibit significant stromal infiltration and cytologic atypia. Because malignant tumors are rare, people frequently confuse them for other types of cancer [4, 5]. BTs are primarily solid on imaging and histopathological examination; however, in up to 30% of cases, pure cystic and mixed solid and cystic forms are recorded in cases of connection with another surface epithelial disease on the same ovary, primarily mucinous tumors [6].

Benign BTs are treated with surgery alone, without chemotherapy, and typically have a great prognosis. Patients with advanced stage disease or residual disease after surgery may be considered for adjuvant chemotherapy in the treatment of borderline BTs. The mainstay of treatment for MBTs is chemotherapy because of their dismal prognosis. To direct treatment and surgical care, a precise preoperative identification of benign and borderline/malignant lesions is necessary [4]. Given the rarity of the disease, we would like to present one case that showed discrepancy between clinical appearance and pathologic examination in benign BT.

## 2. Case Presentation

**Figure 1:**
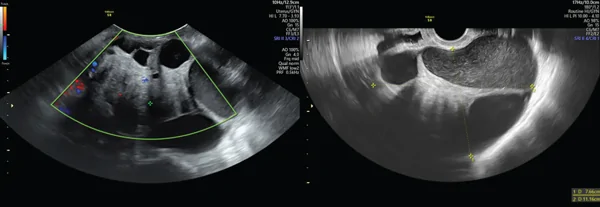
US exam.

Referred from Mitra Plumbon Hospital Cirebon, West Java, a 69‐year‐old woman (parity one, no abortion) with chief complaint abdominal enlargement and abdominal pain 5 months before admission. Patient had weight loss around 10 kg within 2 months. The sized was reported as big as a hand fist with now enlarged as big as a volleyball. Abdominal discomfort was described in the patient, with no complaints of pain. Patient had already menopause approximately 15 years ago. She had no history of surgery and malignancy in the family. The physical examination showed normal vital sign. Abdomen was palpable with mass sized 10 ×10 cm with limited mobility. Vaginal examination presented vulva and vagina within normal limit with palpable adnexal mass sized 10×10 cm with no prominent bulging of Douglas pouch. On ultrasound examination, IOTA simple rules marked with inconclusive result with benign marker (acoustic shadow and no color Doppler projection) and malignant marker (solid part) present. Ca‐125 presurgery was 84.4 u/mL. Risk of malignancy index (RMI)‐2 score was used to assess malignancy. (Figure 1) The RMI‐2 score that consist of ultrasound feature such as solid part and multilocular cyst (4) and postmenopausal women (4). The result was ultrasound feature times postmenopausal status times CA‐125 (4 × 4 × 84.4 = 1350). Ascites was not found in this patient. Chest x‐ray showed no abnormalities.

**Figure 2:**
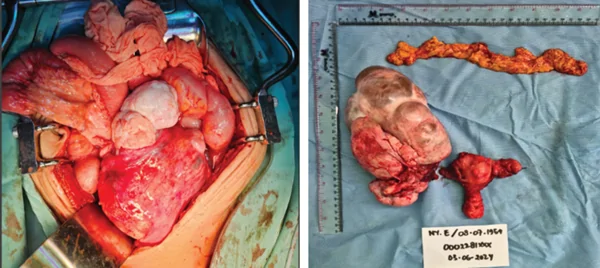
Intraoperative findings.

There were no abnormalities in laboratory examination. The patient had abdominal CT scan from the referral hospital. It showed multilobulated cystic lesion within the pelvic extended to lower abdominal and bladder. A diagnosis of ovarian tumor was established; the patient had undergone abdominal laparotomy with total hysterectomy, bilateral salpingo‐oophorectomy and surgical staging. After peritoneum was opened, a grayish‐white solid mass was found adhered to the ascending colon. The mass was originated from right ovary. The left adnexa and uterus are within normal limits. On exploration, it was found that liver, spleen, and lymph nodes were within normal limits. The mass was adhered to the omentum; we decided to do omentectomy for debulking purposes. (Figure 2).

**Figure 3:**
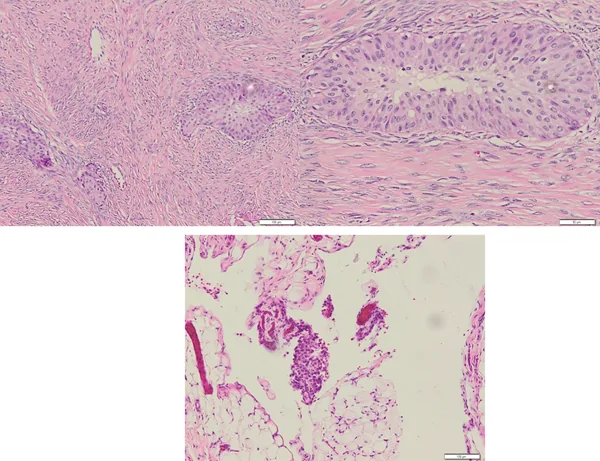
Histopathology findings of the right ovary and omentum.

Histopathology result came 2 weeks after surgery. The initial histopathologic examination of the right ovarian mass revealed nests of transitional type epithelial cells embedded in dense fibrous stroma. However focal areas demonstrated epithelial proliferation with moderate nuclear atypia and increased mitotic activity, raising suspicion for MBT. In addition, microscopic evaluation of the omentectomy specimen showed small epithelial cell clusters morphologically similar to those seen in the ovarian tumor. Because these clusters were located within fibrous tissue and appeared detached from the main tumor mass, the possibility of metastatic involvement to the omentum could not be excluded. (Figure 3).

Based on these findings, the preliminary diagnosis was reported as MBT with suspected omental metastasis. Due to this diagnostic uncertainty, immunohistochemical (IHC) studies were performed to clarify whether the omental lesion represented true metastatic tumor or reactive/entrapped epithelial elements.

**Figure 4:**
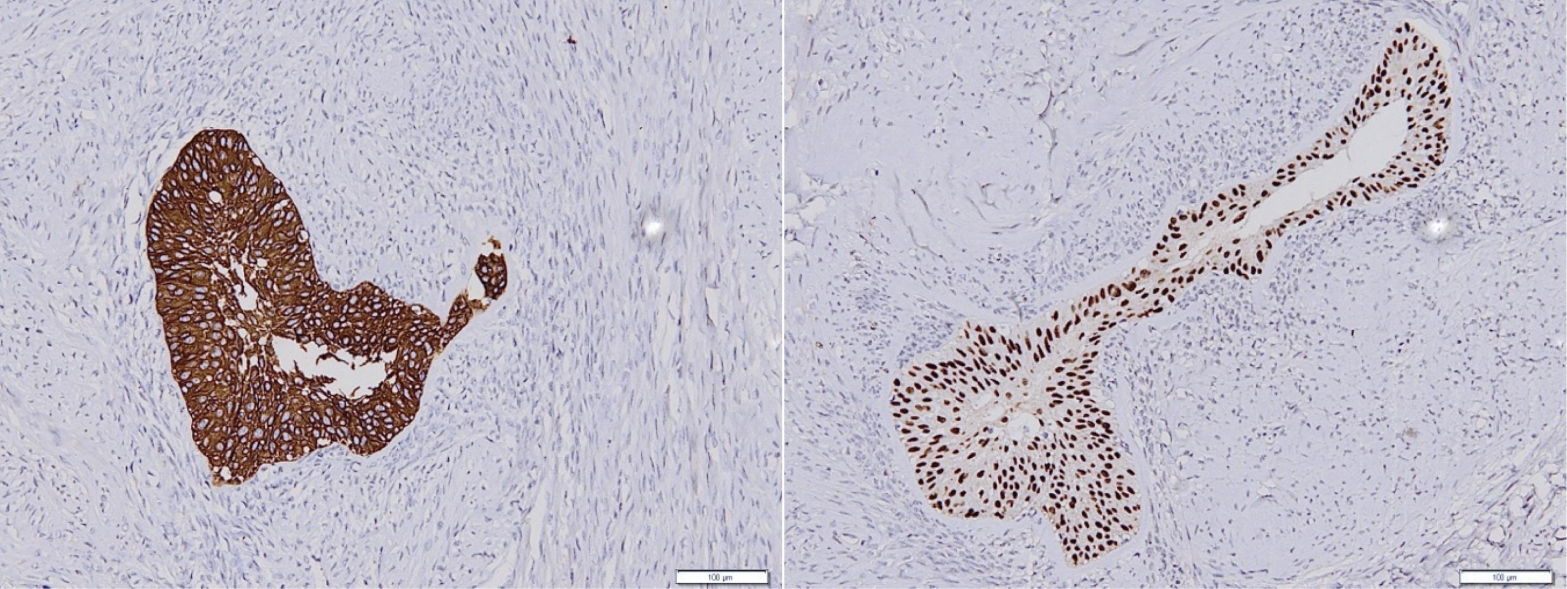
Immunohistochemistry in the ovary (CK7 and p63).

**Figure 5:**
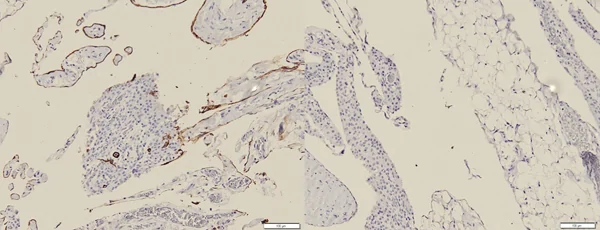
Immunohistochemistry in omentum (CK7 and p63).

There was tumor part suspected metastasis in the omentum. To ensure the potential tumor in the omentum. IHC examination was performed using CK7 and p63 which showed positivity in the ovary but negative in omentum. (Figures 4 and 5) These findings supported the diagnosis of benign BT.

## 3. Discussion

In 2020, ovarian cancer ranked third among gynecological cancers worldwide. Over 90% of cases of ovarian cancer are of ovarian carcinoma, the most common kind of the disease [7]. BTs are an extremely uncommon epithelial neoplasm of the ovary, making up 1.4%–2.5% of all ovarian tumors. Postmenopausal women are typically affected, and the majority of them (approximately 95%) are benign [8]. In our study, the patient characteristics were postmenopausal and 69 years old. As an illustration, a prior study found that patients′ average age was 65. The age of presentation varies depending on the histologic type: mucinous and endometrioid carcinomas occur at 55 years, clear‐cell carcinomas at 56 years, and serous carcinomas at 65 years. MBTs typically show up at a lower stage than serous carcinoma. MBT shares many symptoms with other types of epithelial ovarian carcinomas. Abdominal pain, adnexal, pelvic or abdominal mass, abdominal distention, and vaginal hemorrhage were the most often reported symptoms. Furthermore, in our patients, the symptoms were similar to those commonly associated with malignancy (abdominal pain and weight loss) [1].

Ultrasound examination showed multiloculated cystic mass without the presence of ascites and solid mass. It also showed an acoustic shadow which corresponded to IOTA (International Ovarian Tumor Analysis) B3, and M4 (presence of solid mass) with the result of inconclusive ovarian tumor. On the contrary, we also used RMI‐2 score for the diagnosis. The result was 1350, which showed malignancy for menopausal women. For MBT, there is not a known serological tumor marker. In 30%–75% of patients with MBT, CA‐125 levels are increased; however, there is no correlation between CA‐125 levels and tumor burden [1].

Microscopically, when there is a clear‐cut invasive carcinoma in the context of benign or borderline BT, an MBT diagnosis is justified. The malignant component′s tumor cells are organized into branching trabeculae, solid sheets, or nests with asymmetrical shapes. Pleomorphic hyperchromatic nuclei, rapid mitotic activity, and desmoplastic stromal response are among their high‐grade characteristics. Occasionally, the tumor may have cystic regions surrounded by multilayered epithelium, which is similar to invasive urothelial carcinoma. Mucinous, undifferentiated, or mixed components may be present, but transitional or squamous cell carcinoma is typically the only or predominant malignant component [1].

BTs that are grossly benign are well‐defined, with a cut surface that is gray, white, or slightly yellow, and can be firm or fibromatous. Occasionally, a calcific deposit causes the tissue to become grittier. Benign BT usually measured less than 2 cm with white or pale–yellow cut surfaces [5]. In contrast to our case, the size was 18.5 cm with solid mass. The hallmarks of borderline BTs are cystic, unilocular, or multilocular masses that resemble cauliflower‐like papillomatous growths that protrude into one or more locules. MBTs typically lack any distinguishing characteristics and might be solid or cystic with mural nodules [9].

IHC was performed using CK7 and p63 to further characterize the epithelial component and confirm the nature of suspected omental involvement. CK7 is a cytokeratin marker typically expressed in Müllerian‐derived epithelial tumors, including BTs, whereas p63 is a nuclear marker strongly expressed in transitional (urothelial‐like) epithelial cells, which are characteristic of BTs. In this case, both CK7 and p63 showed diffuse positivity in the ovarian tumor, confirming its transitional epithelial differentiation. (Figure 4) However, the omental specimen was negative for both markers. The absence of CK7 and p63 expression in the omentum excluded metastatic BT and suggested that the previously observed epithelial clusters represented nonneoplastic reactive tissue rather than true metastatic spread. Therefore, the lack of stromal invasion and absence of IHC confirmation of metastasis ultimately supported the final diagnosis of Benign BT.

A significant discrepancy was observed between the preoperative RMI‐2 score and the final histopathologic diagnosis. The patient′s RMI‐2 score was 1350, far exceeding the malignancy threshold of 200 and therefore suggestive of ovarian carcinoma. This elevated score was driven by postmenopausal status, tumor size, ultrasound solid components, and elevated CA125. However, final histopathology with IHC confirmation demonstrated a benign BT. This case highlights an important clinical limitation of RMI: although highly sensitive for epithelial ovarian carcinoma, it lacks specificity for rare tumors such as BTs. Large tumor size, solid components, and mild CA125 elevation may falsely elevate RMI in benign cases. BT of the ovary is exceedingly rare and the diagnosis requires the presence of definitive stromal invasion in the background of benign or borderline BT on histologic examination. Ample tissue sampling and careful microscopic observation should be sufficient to reach the correct diagnosis in most cases. The understanding of BT is still limited because of its rarity. Most of our current knowledge of BT is based on case reports and small case series [4]. This case illustrates that definitive diagnosis of BT subtypes relies on meticulous histopathologic evaluation and, when necessary, IHC rather than clinical risk indices alone.

The majority of BT can be surgically removed. Their highly confined form makes them easily identifiable and usually has little effect on the surrounding tissue. If symptoms exist, surgical excision is frequently curative and will eliminate them. Although MBTs have the potential to spread to other structures and impact surrounding tissue, these occurrences are so uncommon that a conventional treatment has not yet been created. If detected at an early stage, even MBTs are typically candidates for total surgical removal.

## 4. Conclusion

Ovarian BT is extremely uncommon, and the diagnosis necessitates the presence of a clear stromal invasion against a background of benign or borderline BT on histologic analysis. In most circumstances, adequate tissue sampling and meticulous microscopic inspection should be enough to determine the right diagnosis. This case highlights the importance of meticulous histopathologic evaluation and the value of immunohistochemistry when the histopathologic findings are equivocal or metastatic involvement is suspected. In this case, negative CK7 and p63 expression in the omental specimen helped exclude metastatic BT and supported the final diagnosis of benign BT.

## Author Contributions

All authors made a significant contribution to the work reported, whether that is in the conception, study design, execution, acquisition of data, analysis and interpretation, or in all these areas; took part in drafting, revising, or critically reviewing the article.

## Funding

No funding was received for this manuscript.

## Disclosure

All authors have read and approved the final version of the manuscript and had full access to all of the data in this study and take complete responsibility for the integrity of the data and the accuracy of the data analysis. The authors gave final approval of the version to be published; have agreed on the journal to which the article has been submitted; and agree to be accountable for all aspects of the work. The lead author (Sulaeman Andrianto Susilo) affirms that this manuscript is honest, accurate, and transparent account of the study being reported; that no important aspects of the study have been omitted; and that any discrepancies from the study as planned have been explained.

## Ethics Statement

All procedures of the research conformed to the Declaration of Helsinki and the Institutional and/or National Research Council ethical standards. Written informed consent was obtained from the patient for publication of this case report and the accompanying images. Writing and publishing this case report was approved by the Hasan Sadikin Central General Hospital.

## Consent

Written informed consent was obtained from the patient for publication of this case report and the accompanying images. A copy of the written consent is available upon request.

## Conflicts of Interest

The authors declare no conflicts of interest.

## Data Availability

The data that support the findings of this study are available on request from the corresponding author. The data are not publicly available due to privacy or ethical restrictions.
